# Glucose Transporters and Virulence in *Leishmania mexicana*

**DOI:** 10.1128/mSphere.00349-18

**Published:** 2018-08-01

**Authors:** Xiuhong Feng, Khoa D. Tran, Marco A. Sanchez, Hakima Al Mezewghi, Scott M. Landfear

**Affiliations:** a Department of Molecular Microbiology and Immunology, Oregon Health and Sciences University, Portland, Oregon, USA; University of Texas Southwestern

**Keywords:** *Leishmania*, endoplasmic reticulum, gene knockouts, glucose transporter, suppressor, virulence

## Abstract

Glucose transporters are important for viability and infectivity of the disease-causing amastigote stages of Leishmania mexicana. The Δ*gt1-3* null mutant, in which the 3 clustered glucose transporter genes, *GT1*, *GT2*, and *GT3*, have been deleted, is strongly impaired in growth inside macrophages *in vitro*. We have now demonstrated that this null mutant is also impaired in virulence in the BALB/c murine model of infection and forms lesions considerably more slowly than wild-type parasites. Previously, we established that amplification of the *PIFTC3* gene, which encodes an intraflagellar transport protein, both facilitated and accompanied the isolation of the original Δ*gt1-3* null mutant generated in extracellular insect-stage promastigotes. We have now isolated Δ*gt1-3* null mutants without coamplification of *PIFTC3*. These amplicon-negative null mutants are further impaired in growth as promastigotes, compared to the previously described null mutants containing the *PIFTC3* amplification. In contrast, the GT3 glucose transporter plays an especially important role in promoting amastigote viability. A line that expresses only the single glucose transporter GT3 grows as well inside macrophages and induces lesions in animals as robustly as do wild-type amastigotes, but lines expressing only the GT1 or GT2 transporters replicate poorly in macrophages. Strikingly, GT3 is restricted largely to the endoplasmic reticulum in intracellular amastigotes. This observation raises the possibility that GT3 may play an important role as an intracellular glucose transporter in the infectious stage of the parasite life cycle.

**IMPORTANCE** Glucose transport plays important roles for *in vitro* growth of insect-stage promastigotes and especially for viability of intramacrophage mammalian host-stage amastigotes of Leishmania mexicana. However, the roles of the three distinct glucose transporters, GT1, GT2, and GT3, in parasite viability inside macrophages and virulence in mice have not been fully explored. Parasite lines expressing GT1 or GT2 alone were strongly impaired in growth inside macrophages, but lines expressing GT3 alone infected macrophages and caused lesions in mice as robustly as wild-type parasites. Notably, GT3 localizes to the endoplasmic reticulum of intracellular amastigotes, suggesting a potential role for salvage of glucose from that organelle for viability of infectious amastigotes. This study establishes the unique role of GT3 for parasite survival inside host macrophages and for robust virulence in infected animals.

## Abstract (precis)

Glucose transport plays important roles for *in vitro* growth of insect-stage promastigotes and especially for viability of intramacrophage mammalian host-stage amastigotes of Leishmania mexicana. However, the roles of the three distinct glucose transporters, GT1, GT2, and GT3, in parasite viability inside macrophages and virulence in mice have not been fully explored. Parasite lines expressing GT1 or GT2 alone were strongly impaired in growth inside macrophages, but lines expressing GT3 alone infected macrophages and caused lesions in mice as robustly as wild-type parasites. Notably, GT3 localizes to the endoplasmic reticulum of intracellular amastigotes, suggesting a potential role for salvage of glucose from that organelle for viability of infectious amastigotes. This study establishes the unique role of GT3 for parasite survival inside host macrophages and for robust virulence in infected animals.

## INTRODUCTION

*Leishmania* organisms are kinetoplastid parasites that live as extracellular flagellated promastigotes inside the sand fly vector and as nonmotile amastigotes that reside inside acidified phagolysosomal vesicles of the mammalian host macrophages. Glucose is a fuel of central importance in the promastigotes (1–6), which are exposed to high sugar concentrations in the gut of the insect vector following ingestion of plant sap (7). Although glucose is thought to be present at relatively lower levels inside the macrophage parasitophorous vacuole (8, 9), amastigotes are nonetheless dependent upon this nutrient for robust growth. Specifically, glucose transporter (GT) null mutants of Leishmania mexicana that are deficient in uptake of glucose and other hexoses are strongly impaired in growth and survival within murine macrophages (4, 10, 11). Furthermore, glucose and its metabolism via enzymes of the tricarboxylic acid cycle have been proposed to be required for anapleurotic synthesis of glutamate, the major free amino acid in promastigotes and amastigotes, thus implying a critical nutritional role for this sugar (6, 12). Hence, even though glucose and its derivatives (13) are taken up and metabolized less robustly in amastigotes than in promastigotes (12), they are nutrients of central importance for *Leishmania* species, especially in the intracellular amastigote stage, and glucose transporters play the key role in delivering these critical nutrients.

**FIG 1:**
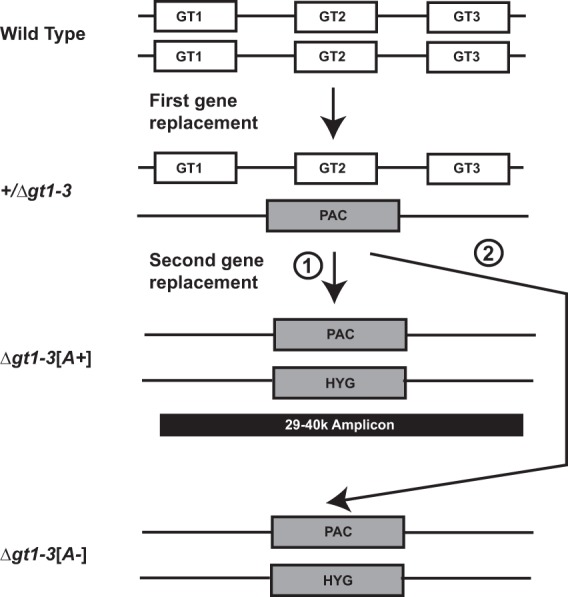
Arrangement of GT genes and generation of the Δ*gt1-3* null mutant that spans this locus. Both alleles of the *GT1*, *GT2*, and *GT3* genes (GT1, GT2, and GT3) are shown on two homologous chromosomes. The first homologous gene replacement employed the puromycin acetyltransferase (PAC) selectable marker to generate the +/Δ*gt1-3* heterozygous deletion mutant, and the second homologous gene replacement employed the hygromycin (HYG) selectable marker to generate the null mutant. During the second targeted gene replacement, a 40-kb linear amplicon arose originating from chromosome 29 (arrow marked with a circled numeral 1; 29-40k amplicon, black rectangle) and encompassing 14 open reading frames. Alternatively, the null mutant lacking the 29-40k amplicon could be generated using the modified protocol described in the text (arrow marked with circled numeral 2). (Modified from Fig. 1A of reference 16 with permission of the publisher.)

In L. mexicana and other *Leishmania* species, a cluster of three genes encodes related but distinct glucose transporter isoforms, GT1, GT2, and GT3 (Fig. 1). Another related but relatively divergent permease, GT4 (14), can transport hexoses with low affinity and induce measurable transport of glucose in parasites when its gene, which is unlinked to the *GT1-GT3* locus, is amplified on an episomal element (11). However, it is not clear whether glucose is a physiologically relevant substrate for the GT4 permease when it is expressed at wild-type (WT) levels. Since promastigotes can survive, albeit with lower rates of growth, in the absence of glucose by oxidizing amino acids (15), it was possible to generate both the Δ*gt1-3* null mutant (4), spanning the *GT1*-to-*GT3* gene cluster, and the Δ*gt1-4* null mutant (16), which was deficient in all 4 GT permeases. Nonetheless, generation of the Δ*gt1-3* null mutant occurred at low frequency and was accompanied by amplification of a linear 40-kb segment of chromosome 29, designated the 29-40k amplicon (16) (Fig. 1; scheme 1, Δ*gt1-3[A+]*). Indeed, generation of the Δ*gt2* single gene null mutant, in which the gene encoding the most abundantly expressed of the 3 glucose permeases, GT2, was especially difficult and was initially achieved only by first stably transfecting the parasites with an open reading frame (ORF) encompassed within the 29-40k amplicon. Curiously, the crucial ORF that promoted the ability to generate the Δ*gt2* null mutant encoded an intraflagellar transport protein, PIFTC3 (16). For Δ*gt2* null mutants, the PIFTC3-containing amplicon could be lost without compromising viability of the null mutant. These observations led to a model whereby amplification of the *PIFTC3* gene promotes the ability to generate the Δ*gt2* null mutant but is not required for its long-term survival. Hence, *PIFTC3* was designated a “transient suppressor” of the Δ*gt2* null mutant, although it is not currently known at the molecular or metabolic level how amplification of this gene promotes retrieval of this null mutant.

In this study, we have further investigated glucose transporter null mutants by (i) interrogating the role of the *PIFTC3* gene as a transient suppressor for GT gene knockouts and (ii) dissecting the function of the individual GT1, GT2, and GT3 transporter isotypes by generating and characterizing double gene knockouts that express only one remaining GT gene. We find that amplification of the *PIFTC3* gene is associated with increased growth of Δ*gt1-3* glucose transporter null mutants as promastigotes but not as amastigotes, thus explaining its ability to facilitate isolation of the slowly growing Δ*gt1-3* null mutant in promastigotes. In contrast, the GT3 permease plays an especially important role in intracellular amastigotes, where expression of this single glucose transporter supports full viability of this infectious life cycle stage, while expression of either GT1 or GT2 alone leads to poor intramacrophage replication.

## RESULTS

### Isolation of Δ*gt1-3* null mutants lacking the 29-40k amplicon.

**FIG 2:**
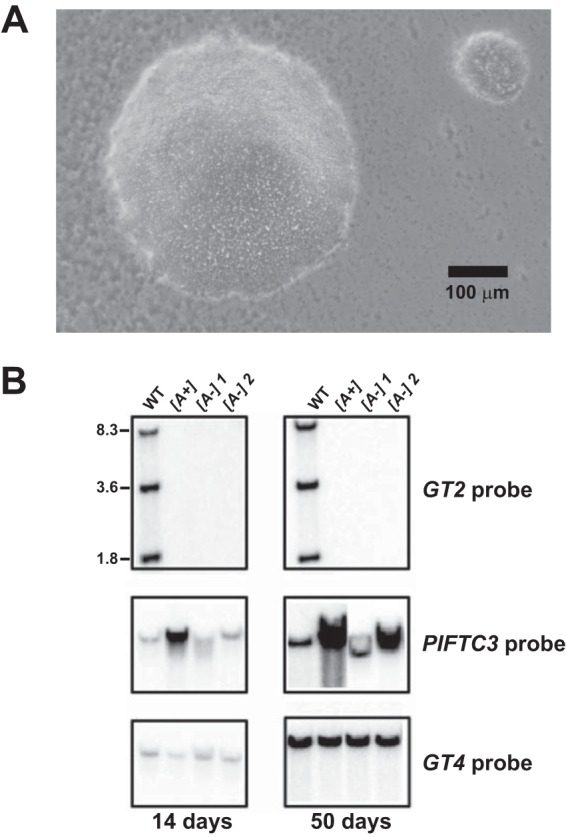
Isolation of Δ*gt1-3[A-]* null mutants lacking the 29-40k amplicon. (A) Microscopic image of an agar plate containing hygromycin-resistant colonies from the second round of targeted gene replacement (see Fig. 1, route 2). Two colonies are shown, of which the one on the top right (diameter, ~130 µm) represents the very small colonies from which Δ*gt1-3[A-]* null mutants were isolated. (B) Genomic Southern blot assays confirming the isolation of Δ*gt1-3[A-]* null mutants and demonstrating the emergence of the 29-40k amplicon in one such line (marked *[A-]2*) after 50 days in culture. Genomic DNA from wild-type (WT) L. mexicana and Δ*gt1-3* null mutants initially either containing (*[A+]*) or not containing (*[A-]*) the 29-40k amplicon was digested with EcoRI/BglII to release the *GT1*, *GT2*, and *GT3* genes as separate fragments. The blot was hybridized with probes for the *GT2* (top panel), *PIFTC3* (middle panel), and *GT4* (bottom panel, loading control) genes. Note that substantial sequence identity between *GT1*, *GT2*, and *GT3* allows the *GT2* probe to hybridize to all three genes. The blot on the left represents clonal isolates that had been passaged in liquid culture for 14 days, and the blot on the right represents the same isolates passaged for 50 days. The intensity of the *PIFTC3* band increased substantially over the wild-type level in the Δ*gt1-3[A-]2* line following 50 days in culture.

Since it was previously shown that Δ*gt2* null mutants could survive expulsion of an amplified *PIFTC3* gene once the null mutants had been generated (16), we asked whether null mutants of the entire gene cluster, Δ*gt1-3*, could be isolated *de novo* without a *PIFTC3*-containing amplicon by employing some modifications of the original selection procedure. Knockout of the GT1 to -3 cluster was repeated using two sequential targeted gene replacements (Fig. 1), but transfectants were plated on semisolid RPMI medium, adjusted to pH 7.0, with hygromycin, and plates were incubated at 27°C for extended periods of time, at least 3 weeks. This treatment allowed very small colonies of ~0.05- to 0.2-mm diameters (Fig. 2A, colony on right) to emerge, not seen in the standard transfection protocols employing shorter ~2-week incubations. Adjusting the initial pH of the plating medium to 7.0 was also important for obtaining the small colonies, as they did not grow well in the medium employed for conventional transfections where the initial pH was 7.4. Poor growth on pH 7.4 medium was likely due to the fact that these null mutants do not take up glucose and metabolize it to various acidic end products (17). Consequently, unlike wild-type parasites, these null mutants increase the pH of the surrounding medium, resulting in impaired growth. The adjustment of plates to pH 7.0 counterbalanced this growth inhibition by retarding the increase in pH. Southern blot analyses of genomic DNA isolated from several of these small colonies (Fig. 2B) demonstrated the absence of the *GT1*, *GT2*, and *GT3* genes and also the absence of an amplified *PIFTC3* gene (Fig. 1, Δ*gt1-3[A-]*, and 2B, 14-day panel at the left, *[A-]1* and *[A-]2*, where *[A-]* indicates the absence of the 29-40k amplicon). In contrast, DNA isolated from larger colonies (Fig. 2A) was also deleted in all three *GT* genes but had markedly more intense hybridization to the *PIFTC3* probe (Fig. 1, Δ*gt1-3[A+]*, and 2B, *[A+]*), indicating the presence of the 29-40k amplicon that contains the *PIFTC3* gene (16).

**FIG 3:**
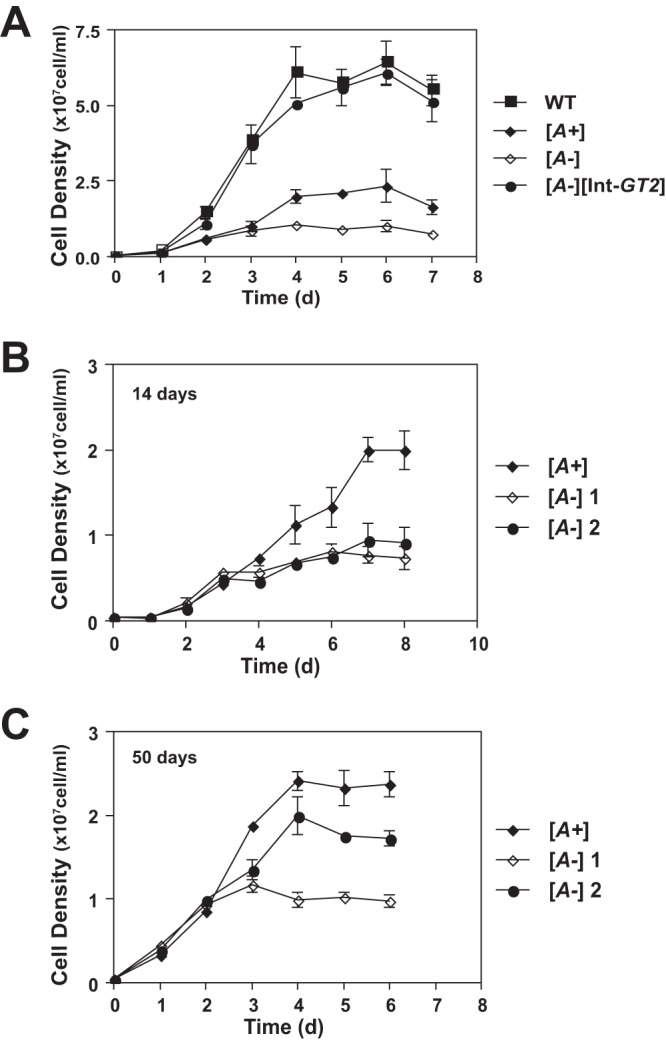
Growth phenotypes of Δ*gt1-3[A+]* (*[A+]*) and Δ*gt1-3[A-]* (*[A-]*) null mutants as promastigotes in liquid culture. (A) Growth of wild-type (WT), Δ*gt1-3[A+]*, Δ*gt1-3[A-]*, and Δ*gt1-3[A-]* [Int-*GT2*] lines in RPMI medium. Parasites were inoculated at a density of 5 × 10^5^ cells/ml, and growth was followed for 7 days. Cell densities represent the averages and standard deviations from 3 replicate counts. (B and C) Growth curves were also measured for one *[A+]* and two *[A-]* clonal Δ*gt1-3* null mutant lines after passaging the lines for 14 days (B) or 50 days (C).

Both current (Fig. 3A) and previous (16) studies on Δ*gt1-3* null mutants containing the 29-40k amplicon (Δ*gt1-3[A+]*) demonstrate that promastigotes of these mutants grow more slowly and to a lower stationary-phase cell density (~2 × 10^7^ cells/ml) than wild-type parasites (~6 × 10^7^ cells/ml) in RPMI 1640 medium containing 10 mM glucose and 10% fetal calf serum (Fig. 3A, *[A+]*). Complementation of the null mutant with an integrated *GT2* gene (Fig. 3A, [*A-*][Int-*GT2*]) restores wild-type growth. In contrast, amplicon-negative Δ*gt1-3[A-]* mutants (Fig. 3A, *[A-]*) grow even more slowly and to a lower stationary-phase density (~0.5 × 10^7^ to 1 × 10^7^ cells/ml) than the amplicon-containing Δ*gt1-3[A+]* null mutants. Hence, the presence of the 29-40k amplicon promotes growth of *Δgt1-3* null mutants, both in semisolid medium (larger, more rapidly growing colonies for *[A+]* lines) and in liquid medium (higher rate of growth and higher stationary-phase cell density for *[A+]* lines).

Notably, the growth properties of one of the five *[A-]* mutants examined changed as parasites were passaged over time. The growth curves for two of these mutants (Fig. 3B, *[A-]1* and *[A-]2*) were initiated after 14 days of passage and, alternatively, following 50 days of passage in liquid medium (Fig. 3C). At this later time, clonal line Δ*gt1-3[A-]2* had begun to exhibit growth properties similar to the amplicon-containing *[A+]* line and reached a stationary-phase density of ~2 × 10^7^ cells/ml. This line had acquired the 29-40k amplicon, as demonstrated by the increased intensity of the *PIFTC3*-hybridizing band in a Southern blot (Fig. 2B, right panels). These data indicate that (i) Δ*gt1-3[A-]* mutants are viable but exhibit reduced growth compared to the *[A+]* lines; (ii) the linear 29-40k amplicon can be generated spontaneously in the *[A-]* lines (18); and (iii) once generated, the 29-40k amplicon provides a growth advantage that allows promastigotes containing this extrachromosomal element to outgrow amplicon-negative parasites in culture. These results also explain why the initial Δ*gt1-3* null mutants (4) isolated under standard plating conditions contained the 29-40k amplicon (16) and that isolation of amplicon-negative null mutants was achieved only following extended growth on plates at the optimal more-acidic pH.

### Growth of Δ*gt1-3[A+]* and Δ*gt1-3[A-]* null mutants in murine macrophages.

**FIG 4:**
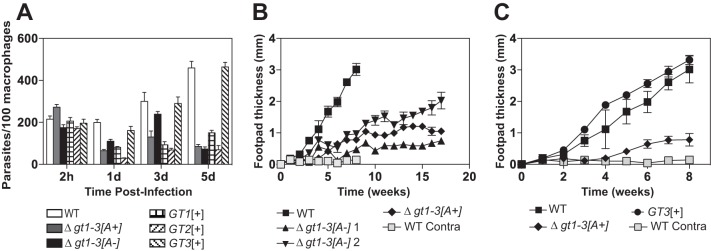
Infectivity and virulence of glucose transporter null mutants. (A) Survival of glucose transporter null mutants in primary bone marrow macrophages. Murine macrophages were infected with stationary-phase promastigotes of the indicated parasite lines, and intracellular parasites were enumerated at 2 h and 1, 3, and 5 days after infection. Each sample was counted as 3 separate infections, and the values graphed represent the averages and standard deviations. (B) Lesion size (change in footpad thickness) in BALB/c mice infected with wild-type L. mexicana and Δ*gt1-3* null mutants that either have (*[A+]*) or do not have (*[A-]*) the 29-40k amplicon. Two clonal isolates of the Δ*gt1-3[A-]* line were employed and designated *[A-]1* and *[A-]2*. Five mice each were infected in one hind footpad with 5 × 10^6^ stationary-phase promastigotes of each line. Footpad thickness was measured at the time of infection, and the increase in this value was measured each week. Values plotted represent the average and standard deviations for each cohort of 5 mice. Light gray squares represent measurements of the contralateral noninfected footpad thickness for wild-type parasites. For the Δ*gt1-3[A+]* clone, the infection experiments were repeated a second time with the same results. (C) Lesion size in BALB/c mice infected with wild-type, Δ*gt1-3[A+]*, and *GT3[+]* lines. All data represent a separate experiment from that shown in panel B.

Previous studies (4, 11) have established that Δ*gt1-3[A+]* null mutants are strongly impaired for growth and survival within murine macrophages. Since the *[A-]* mutants exhibited a reduced growth phenotype as promastigotes compared to *[A+]* promastigotes, we wished to determine whether the *[A-]* null mutants might also be more strongly impaired than *[A+]* null mutants for growth as amastigotes inside macrophages. Infections of murine primary bone marrow-derived macrophages with wild type, Δ*gt1-3[A+]*, and Δ*gt1-3[A-]* null mutant cells showed no substantial differences in internalization of parasites 2 h postinfection (Fig. 4A), and the two knockout lines have similarly reduced numbers of intracellular parasites at day 5 postinfection. In contrast, wild-type parasites at day 5 replicated to ~5-fold-higher levels than that of either null mutant line. Hence, the *[A-]* null mutants are not more compromised for growth than the *[A+]* null mutants in primary murine macrophages, but both are strongly impaired compared to wild-type amastigotes.

### Infections of BALB/c mice with Δ*gt1-3[A+]* and Δ*gt1-3[A-]* null mutants.

To define the virulence phenotype of Δ*gt1-3[A+]* and Δ*gt1-3[A-]* genotypes, each line was used to infect footpads of BALB/c mice (Fig. 4B). Wild-type L. mexicana induced lesions of ~3-mm thickness within 8 weeks of inoculation. In contrast, both *[A+]* and *[A-]* knockout lines generated much smaller lesions with a considerably slower time course, indicating significantly attenuated virulence. Two different clonal *[A-]* knockout lines produced somewhat different kinetics of lesion formation, either slightly faster (downward triangles, Fig. 4B) or slightly slower (upward triangles, Fig. 4B) than the single *[A+]* line examined (diamonds, Fig. 4B). In summary, all knockout lines exhibited significantly reduced virulence, but no consistent difference was observed between *[A+]* and *[A-]* lines. The observation that three independently derived Δ*gt1-3* null mutants, both with and without the amplicon, are quantitatively impaired in virulence also supports the conclusion that this phenotype is due to loss of this multigene locus and not to another “off-target” genetic lesion.

It is notable that infections of BALB/c mice with one of our Δ*gt1-3[A+]* mutants, carried out by our colleagues in the laboratory of Malcolm McConville, failed to produce lesions and thus indicated an avirulent phenotype (19). Those infections were performed by injecting parasites at the base of the tail, as opposed to the infections reported here that employed footpad injections. Hence, the route of infection may affect the virulence phenotype observed for these null mutants, a variable that has been documented to affect parasite virulence in other studies (20).

### Generation and characterization of knockout lines expressing single GT genes.

**FIG 5:**
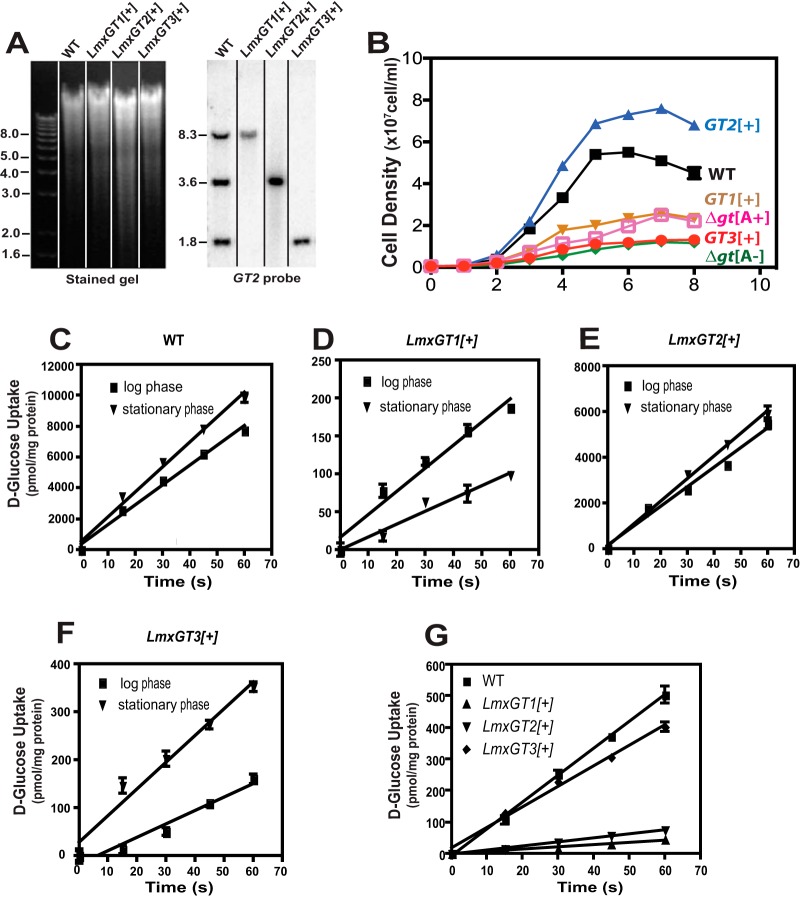
Characterization of parasite lines that retain only a single glucose transporter gene. For each line, 2 of the 3 glucose transporter genes were removed by targeted gene replacement, and the resulting dual knockout was named for the one remaining glucose transporter gene: *GT1[+]*, *GT2[+]*, and* GT3[+]*. (A) Genomic DNA from each line was digested with EcoRI and BglII, separated on an agarose gel, blotted onto a nitrocellulose membrane, and hybridized with the *GT2* ORF (*GT2* probe). Numbers at the left of each image indicate the positions of DNA molecular weight markers, designated in kilobase pairs. The vertical lines separating lanes in the figure indicate that different lanes from the same gel and blot were assembled to remove irrelevant lanes. (B) Cell density was measured as a function of days (d) of growth for each of the parasite lines, indicated to the right. Values plotted represent the averages and standard deviations from 3 replicate measurements. (C to F) Uptake of 100 µM d-[^14^C]glucose was determined over a 60-s time course for promastigotes of the wild type and each single GT expresser line. Each line was examined during both the logarithmic and stationary phases of growth. (G) Uptake of 100 µM d-[^14^C]glucose by axenic amastigotes of wild-type and single-GT expresser lines. For panels C to G, values plotted represent the averages and standard deviations for 3 replicate uptake measurements.

One fundamental question that remains incompletely answered has to do with the potentially distinct functions of GT1, GT2, and GT3 transporters. To address the function of individual GTs, the Δ*gt2/3* (expressing only *GT1*), Δ*gt1/3* (expressing only *GT2*), and Δ*gt1/2* (expressing only *GT3*) dual null mutants were created by targeted gene replacement. Thus, for the Δ*gt2-3* mutant, these adjacent genes were removed by a single step of homologous gene replacement employing the relevant flanking sequences as targets, leaving only the *GT1* gene intact. For the Δ*gt1/3* mutant, the *GT1* and *GT3* genes were removed in two separate steps using distinct homologous integrations, leaving only the *GT2* gene intact. For simplicity, these dual null mutants will be referred to here as the *GT1[+]*, *GT2[+]*, and *GT3[+]* lines, respectively, indicating the single GT gene that they retain. Genomic Southern blots for each of these dual null mutants, confirming the retention of a single GT gene, are shown in Fig. 5A. Growth curves for promastigotes of each dual null mutant grown in glucose-replete (10 mM glucose) RPMI 1640 (Fig. 5B) establish that *GT2[+]* grows as well as or better than wild-type parasites but that the *GT1[+]* and *GT3[+]* lines are strongly impaired in growth. Furthermore, uptake of 100 µM d-[^3^H]glucose by promastigotes of each line is shown in Fig. 5C to F. The *GT2[+]* line transports glucose at rates similar to those of wild-type promastigotes (compare Fig. 5C and E; ~6,000 and ~10,000 pmol/min/mg protein, respectively), but the *GT1[+]* and *GT3[+]* lines exhibit much lower rates of glucose uptake (Fig. 5D and F; ~200 and ~350 pmol/min/mg, respectively). These results confirm that GT2 is the principal glucose transporter in promastigotes. Furthermore, comparison of glucose uptake rates reveals that the function of GT1 is reduced but GT3 is enhanced in stationary-phase parasites compared to logarithmic-phase cells, while GT2 is not significantly affected by promastigote growth phase. These results are consistent with recent studies on regulation of protein expression level for these three permeases as a function of parasite cell density (21).

### GT3 plays a key role in intracellular amastigotes.

Infection of murine primary macrophages revealed that *GT1[+]* and *GT2[+]* amastigotes survived poorly in host cells but that *GT3[+]* survived and replicated as well as wild-type amastigotes (Fig. 4A). This result, which was replicated using two independently generated *GT3[+]* lines, implies that GT3 is the most important glucose transporter for the infectious stage of the life cycle. The observed prominent role for GT3 in infectivity is also consistent with the observation that *GT2* mRNA is markedly downregulated upon transformation of promastigotes to amastigotes (22) and that both the GT1 and GT2 proteins are poorly expressed in intracellular amastigotes compared to promastigotes (21), both of which would limit the role of these permeases in amastigotes.

To investigate the role of GT3 during *in vivo* infections, BALB/c mice were infected with the *GT3[+]* mutant (Fig. 4C, filled circles). In contrast to the various Δ*gt1-3* strains, the *GT3[+]*-induced lesions were robust, measuring ~3 mm by week 8 and exhibiting virulence comparable to wild-type parasites. Thus, expression of the GT3 transporter alone from its endogenous locus is sufficient for full virulence *in vivo* as well as *in vitro*.

Recent studies on GT1 employed a novel assay (21) to demonstrate that expression of this permease is regulated at the level of protein stability. Expression from a single mRNA of a GT1-2A-luciferase fusion protein, in which the 2A peptide induces cotranslational cleavage releasing separate GT1-2A and luciferase peptides, exhibited robust accumulation of luciferase but not of GT1, confirming that the latter protein is degraded in amastigotes while luciferase is stable. The results in Fig. 4 suggest that the GT3 protein may be relatively more stable in the intracellular stage of the parasite life cycle than GT1 and GT2 and thus is able to selectively support viability of amastigotes. Consistent with this interpretation, measurement of rates of uptake for 500 µM d-[^14^C]glucose for each single GT-expressing line in axenic amastigotes (Fig. 5G) indicated that wild-type and *GT3[+]* axenic amastigotes have almost equivalent rates of glucose uptake, whereas uptake by the *GT1[+]* and *GT2[+]* lines was much less robust. This result is consistent with our previous observation that GT1 and GT2 are not stable in intracellular amastigotes.

**FIG 6:**
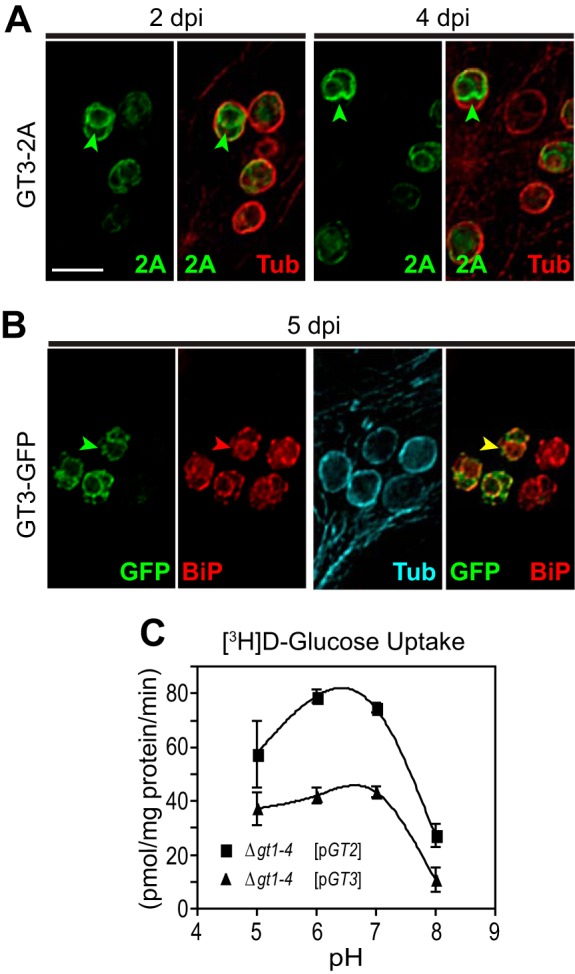
Expression of GT3 in amastigotes. (A) Amastigotes expressing GT3-2A were imaged inside THP-1 host macrophages at 2 and 4 days postinfection (dpi) by deconvolution microscopy to detect the 2A-tagged fusion protein (green) or α-tubulin (red). Arrowheads designate intracellular fluorescence for GT3-2A. Bar, 5 µm. (B) At 5 dpi, intracellular amastigotes expressing the GT3-GFP fusion protein were stained with anti-GFP antibody (green), with antibody to BiP (red), and with antibody to α-tubulin (cyan). Green arrowheads indicate intracellular GT3-GFP, red arrowheads are the BiP marker for the ER, and the yellow arrowhead indicates costaining of GFP and BiP. Bar, 5 µm. (C) Uptake of 100 µM d-[^3^H]glucose by GT2 and GT3 as a function of pH. Rates of uptake were measured in triplicate, and data points represent the means and standard deviations (error bars). Cell lines employed were the glucose transporter null mutant Δ*gt1-4* (16) encompassing either the *GT2* ([pGT2]) or *GT3* ([pGT3]) open reading frame in an episomal expression vector.

To further examine expression of GT3 in intracellular amastigotes, we transfected episomes encoding the GT3-2A-luciferase and the GT3-green fluorescent protein (GFP) fusion proteins into L. mexicana parasites and infected THP-1 macrophages. Immunofluorescence imaging established that GT3-2A (Fig. 6A) is still robustly expressed at 4 days and LxmGT3-GFP (Fig. 6B) is still robustly expressed at 5 days postinfection. These results are consistent with the prominent role for GT3 in supporting amastigote viability observed using the *GT3[+]* strain.

Another potential explanation for the critical role of GT3 in amastigotes could be that this permease might be activated at the acidic pH maintained within the parasitophorous vacuole and thus could mediate enhanced transport inside the vacuole. However, measurement of pH activity profiles for uptake of 100 µM d-[^3^H]glucose demonstrated that both GT2 and GT3 have pH maxima close to neutrality (Fig. 6C), eliminating activation at low pH as a potential reason for the central role of GT3 in amastigotes.

### GT3 is expressed in the ER of amastigotes.

The availability of constructs expressing GT3 fused to either 2A or GFP tags allows determination of subcellular localization of these fusion proteins. Integration of the GT3-2A-luciferase fusion into the *GT3* gene locus was carried out initially to achieve expression of the tagged permease at endogenous levels. However, immunofluorescence images and Western blot assays of these parasites failed to detect the GT3-2A protein, indicating that the endogenously expressed protein is synthesized at too low a level to allow detection by this methodology. Hence, amastigotes overexpressing the GT3-2A protein from an episomal expression vector, as depicted in Fig. 6A, were employed to monitor subcellular localization. Notably, all of the GT3-2A fluorescence appears to be inside the amastigotes, underneath the subpellicular microtubules that are imaged with anti-α-tubulin antibody, suggesting a possible localization to the endoplasmic reticulum (ER). To confirm localization in this organelle, amastigotes expressing the GT3-GFP fusion were costained with both anti-GFP (chicken antibody) and anti-BiP (rabbit antibody) antibodies (Fig. 6B). The GT3-GFP fusion was employed because, unlike GT3-2A (anti-2A is a rabbit antibody), it can be detected with a different secondary antibody from that used to detect BiP. A high degree of overlap between the anti-GFP (green) and anti-BiP (red) staining is apparent, confirming the localization of GT3-GFP to the ER.

In principle, retention of GT3 fusion proteins in the ER could represent an artifact of overexpression, an experimental necessity required by the inability to detect the protein by immunofluorescence when it is expressed at wild-type levels. However, an observation that suggests that the ER is probably the correct localization for GT3 is the presence of the C-terminal sequence KKEM on GT3 but not on GT1 or GT2. This sequence conforms to the (K/X)(K/X)KXX-stop motif (where X is any amino acid and 2 K residues are present at positions −3 and either −4 or −5) proposed for a generic C-terminal ER retention signal for membrane proteins (23, 24) and demonstrated to be required for ER localization of a Ca^2+^-ATPase from Trypanosoma cruzi (25).

### Subcellular localization of GT3 is regulated during growth of promastigotes.

**FIG 7:**
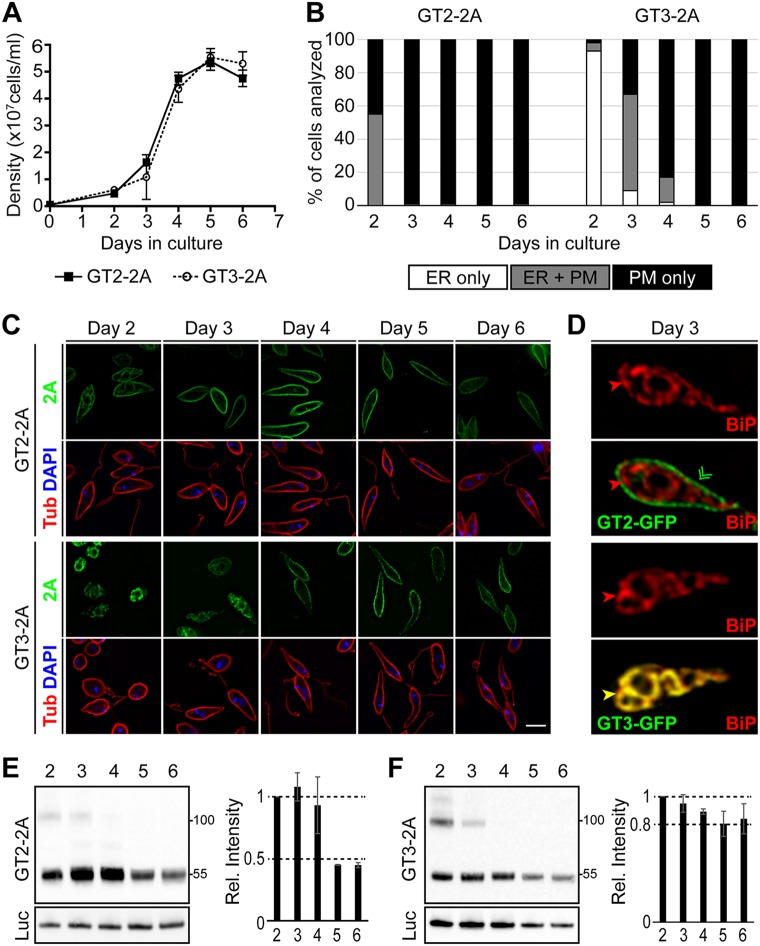
Localization of GT2 and GT3 in promastigotes. (A) Growth curves for wild-type L. mexicana promastigotes expressing the GT2-2A and GT3-2A proteins generated from cotranslational cleavage from luciferase fusion proteins. Cell densities were determined from 2 separate cultures. The cell density of each culture was determined by replicate measurements, which were then averaged, and the mean and range (error bars) of the averages from the 2 experiments were plotted. (B) Promastigotes expressing either GT2-2A or GT3-2A were removed from each culture daily and imaged by deconvolution microscopy employing the anti-2A antibody. Individual cells (>150 for each time point) were scored for localization of the tagged protein in the ER only, the ER plus plasma membrane (PM), or the plasma membrane only, and the percentage of cells in each group was plotted. (C) Fields of parasites prepared as described for panel B were imaged by deconvolution microscopy following staining with antibody to the 2A epitope (green), antibody to α-tubulin (red), and DAPI (blue). Bar, 5 µm. (D) A parasite expressing GT2-GFP (top 2 panels) from a day 3 culture (cell density, ~1 × 10^7^ cells/ml) was imaged using antibody to BiP (red) and to GFP (green). The merged image (second panel from the top) demonstrates localization of the transporter on the cell surface and no overlap with BiP. A parasite expressing GT3-GFP (bottom 2 panels) from a day 3 culture was imaged with antibody to BiP (red) and to GFP (green). The merged image (bottom-most panel) shows yellow color indicating extensive overlap between GT3-GFP and BiP. Bar, 5 µm. (E) Western blot of lysates from parasites expressing GT2-2A, generated from a luciferase fusion construct, at days 2 to 6 of growth. The top panel was developed with anti-2A antibody, and the bottom panel was developed with antiluciferase antibody. Two separate experiments were performed, the intensity of the signals was determined by scanning the developed blot, and the average and range of the two relative intensities at each day were plotted, setting the day 2 intensity to a value of 1. Molecular weight markers in kilodaltons are shown at the right. (F) Western blot as in panel E for parasites expressing GT3-2A. The bands at ~100 kDa on the blots in panels E and F may represent dimers of the transporters that have remained intact during electrophoresis on SDS gels.

Observations in promastigotes expressing GT3-2A also suggest that ER localization is likely not an artifact. Promastigotes monitored in the early stages of a growth curve (Fig. 7A, at densities below ~1 × 10^6^ cells/ml) exhibit restriction of GT3-2A staining to intracellular sites (Fig. 7B and C, days 2 and 3) that costain with BiP (Fig. 7D, bottom 2 panels). Strikingly, as promastigotes reach stationary phase (days 4 to 6), GT3-2A stain relocalizes to the surface of the cell, coincident with the subpellicular microtubules that subtend the plasma membrane and stain with anti-α-tubulin antibody (Fig. 7B and C, days 4, 5, and 6). In contrast, GT2-2A localizes largely to the cell surface in promastigotes that are at either low or high cell density (Fig. 7C and D, top 2 panels). Hence, GT3 subcellular localization is regulated by parasite density or some property associated with cell density. This regulated localization further suggests that ER retention is likely not an artifact of overexpression but is of biological significance.

One intriguing possibility is that ER retention of GT3 could be responsive to glucose levels in the medium, with retention being favored at high glucose concentrations in low-density medium and trafficking to the cell surface occurring when glucose is depleted by uptake and metabolism in high-density cultures. However, we were unable to alter the localization of GT3 in either low- or high-density cultures by either adding or removing glucose or by transferring parasites to conditioned medium derived from either low- or high-density cultures (data not shown). Hence, trafficking of GT3-GFP was not altered by (i) depletion or addition of glucose, (ii) natural depletion of another component of the medium during growth, or (iii) exposure to a component that accumulates in 4-day “conditioned” medium.

In addition, monitoring of GT2-2A (Fig. 7E) and GT3-2A (Fig. 7F) proteins over the course of the promastigote growth curve by Western blotting demonstrated that both proteins continue to be expressed throughout the growth phase, although GT2-2A drops in abundance following prolonged cultivation in stationary phase (days 5 and 6), whereas GT3-2A is somewhat more resistant to this reduction in expression during stationary phase.

### Sequences within GT3 important for localization to the ER.

**FIG 8:**
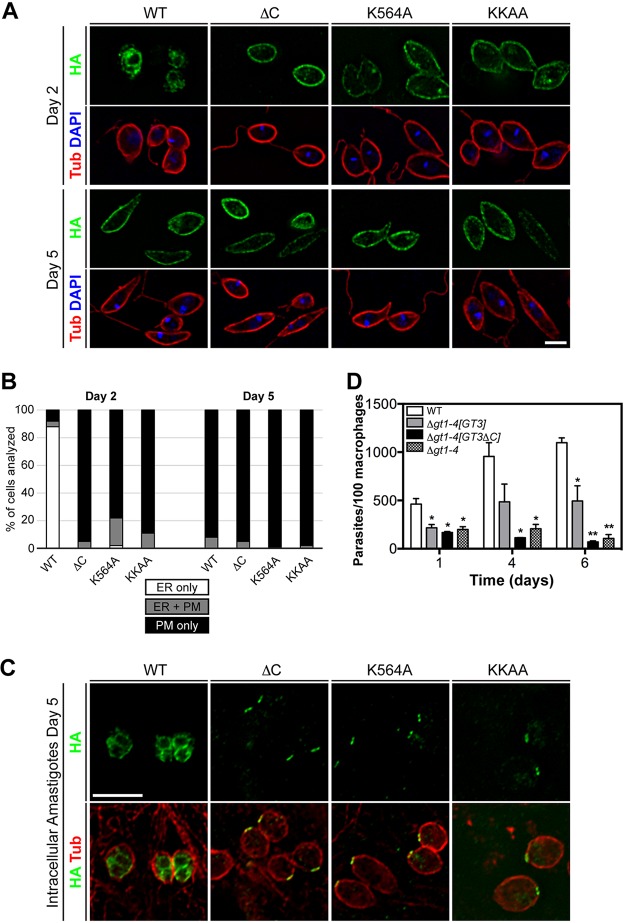
Localization of wild-type and mutant GT3 in promastigotes and amastigotes. (A) Deconvolution immunofluorescence images of promastigotes expressing wild-type (WT) GT3-HA_3_ or the fusion protein that has been truncated immediately before the KKEM motif (ΔC), or for which K564 (K564A) or both K564 and K565 (KKAA) within this motif have been mutated to alanine residues. Parasites were diluted to a density of 10^5^ cells/ml and grown for either 2 or 5 days to achieve a density of <10^7^ cells/ml (day 2) or >5 × 10^7^ cells/ml (day 5), respectively. Images show fluorescence for HA (green), α-tubulin (red), and DAPI (blue). Bar, 5 µm. (B) Quantification of immunofluorescence results for the parasite lines imaged in panel A. For each line, 50 to 70 parasites were scored as having detectable fluorescence in the ER alone (ER only), the plasma membrane only (PM only), or in both locations (ER + PM). (C) Deconvolution immunofluorescence images of amastigotes inside infected THP-1 macrophages at 5 days postinfection, expressing wild-type or mutant versions of GT3-HA_3_. The figure shows HA fluorescence alone (top row) or both HA and tubulin fluorescence (bottom row). Bar, 5 µm. (D) Quantification of intracellular amastigotes for infections of THP-1 macrophages with wild type (WT), Δ*gt1-4* null mutant, and this null mutant complemented with full-length GT3 (*[GT3]*) or with C-terminally truncated GT3 (*[GT3ΔC]*). Data are plotted as intracellular parasites per 100 macrophages for two independent infections displayed as means and standard deviations (error bars). Statistical comparisons (*t* test; *, *P* = 0.05; **, *P* = 0.01) refer to comparisons between each infection and that for wild-type parasites.

To determine whether the KKEM motif in GT3 is required for ER localization, we altered this sequence by (i) mutating K564 to an alanine (K564A mutant), (ii) mutating both K564 and K565 to alanines (KKAA mutant), and (iii) truncating the C terminus by placing a stop codon immediately preceding the KKEM motif (ΔC mutant). All mutants were expressed from an episomal vector as triple-hemagglutinin epitope-tagged (HA_3_) fusion proteins to monitor localization. In contrast to wild-type GT3, each of the 3 mutants localized largely to the plasma membrane rather than the ER in promastigotes cultured at low density, as demonstrated both by immunofluorescence images (Fig. 8A) and by quantification of the percent parasites with staining in the ER only, the plasma membrane only, or both locations (Fig. 8B).

Imaging of amastigotes within infected THP-1 macrophages demonstrated that most or all of the fluorescence from wild-type GT3 was intracellular (Fig. 8C, WT), beneath the microtubule corset that stains with tubulin antibody, presumably reflecting localization to the ER. Hence, like promastigotes at low cell density, amastigotes express little of this permease on their cell surface. In contrast, each of the 3 mutants in the KKEM motif relocalizes, with pronounced fluorescence present on the anterior tip of the amastigote, possibly representing the interface between the flagellar pocket membrane and the pellicular plasma membrane. In addition, there is also punctate intracellular fluorescence to various degrees among different amastigotes. While we do not know why these mutant versions of GT3 traffic to these regions of amastigotes rather than uniformly over the pellicular plasma membrane, all three mutants in the KKEM motif are released from the ER, as they are in promastigotes. The dependency of ER localization upon the KKEM motif in both life cycle forms further suggests that this subcellular localization of GT3 does not simply represent an artifact of overexpression but that the permease is actively targeted to this intracellular organelle.

**FIG 9:**
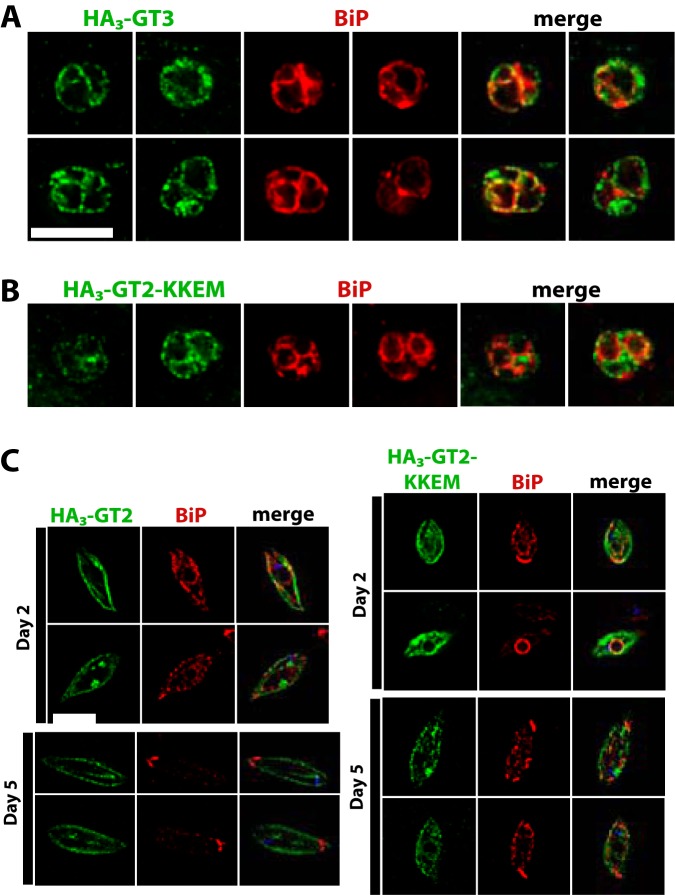
Trafficking of GT2 and GT3 glucose transporters tagged with the HA_3_ epitope at the amino-terminal end. In all images, green represents the secondary immunofluorescence signal of the relevant permease, red represents secondary immunofluorescence corresponding to the ER protein BiP, and “merge” shows the overlap of the green and red signals. (A) Intracellular amastigotes expressing HA_3_-GT3. (B) Intracellular amastigotes expressing HA_3_-GT2-KKEM, where KKEM is the ER targeting motif. (C) Promastigotes expressing HA_3_-GT2 (left panels) and HA_3_-GT2-KKEM (right panels). Images were taken on day 2 (~1 × 10^6^ cells/ml, top panels) and day 5 (~1 × 10^7^ cells/ml, bottom panels) of culture to capture parasites in low and high density. Bars, 6 µm; the bar in panel A also represents the scale for panel B.

To confirm that the KKEM motif is a determinant of ER localization, we created another construct in which the HA_3_ tag was placed onto the N terminus of GT3, where it could not interfere with an adjacent C-terminal KKEM. This N-terminally tagged GT3 also trafficked largely to the ER in amastigotes, as indicated by overlap with BiP fluorescence (Fig. 9A). When the normally plasma membrane-resident GT2 was tagged with the HA_3_ epitope on its N terminus and with the KKEM motif on its C terminus, there were a limited number of amastigotes exhibiting fluorescence at 4 days postinfection, due to the poor stability of the GT2 permease in amastigotes. Those that did have fluorescent amastigotes showed internal signal for the HA_3_::GT2-KKEM construct, but overlap with the BiP signal was poor (Fig. 9B). Similarly, overlap of the HA_3_::GT2-KKEM signal with BiP was not extensive in promastigotes at either low (2 days of culture) or high (5 days of culture) density (Fig. 9C). Hence, while the KKEM signal is essential for targeting of GTs to the ER, it is not sufficient.

To determine whether ER localization of GT3 is functionally important in amastigotes, we quantified infections of THP-1 macrophages with parasites expressing either full-length GT3 or the GT3-ΔC truncated mutant (Fig. 8D). These GT3 variants were expressed in the Δ*gt1-4* null mutant (16), which is deficient in endogenous glucose transporters (deleted for the entire *GT1* to -*3* cluster plus the gene for another low-affinity glucose transporter, *GT4*, which can become amplified over time in a Δ*gt1-3* null mutant [11]), and the infectivity to macrophages was compared to that of wild-type (WT) L. mexicana and to the uncomplemented Δ*gt1-4* null mutant. Wild-type parasites (Fig. 8D, empty bars at left) replicated robustly inside the macrophages, whereas the Δ*gt1-4* null mutants (Fig. 8D, stippled bars at right) entered macrophages and transformed to amastigotes but decreased dramatically in abundance by day 6 of infection. The null mutants complemented with full-length *GT3* (Fig. 8D, black bars) replicated significantly better than the uncomplemented null mutant, but those complemented with the *GT3-ΔC* mutant survived poorly, similarly to the null mutant. Variation in the level of growth of the null mutant complemented with *GT3* was significant between independent replicates, and thus, the difference in growth between amastigotes expressing GT3 and those expressing GT3*-Δ*C did not attain statistical significance at a confidence limit of *P* = 0.05. Nonetheless, the data suggest that the localization of GT3 to the ER is likely to be relevant to its ability to promote viability of amastigotes inside host macrophages.

## DISCUSSION

Previous studies have highlighted the importance of glucose transporters in both insect-stage promastigotes and intracellular amastigotes of L. mexicana (4, 10, 16, 21). However, a number of key questions remain to be resolved concerning the role of this family of proteins throughout the *Leishmania* life cycle. First, the role of the 29-40k amplicon that accompanied deletion of the glucose transporter gene cluster remained uncertain. Second, the functions of individual glucose transporter isoforms for viability of intracellular amastigotes and for virulence in the animal model of disease required further elucidation.

### *Δgt1-3[A-]* null mutants lacking the PIFTC3-containing amplicon.

We have generated by targeted gene replacement Δ*gt1-3[A-]* null mutants that are devoid of the 29-40k amplicon (Fig. 1) that had accompanied the previously described Δ*gt1-3* null mutants (16). The ability to obtain Δ*gt1-3[A-]* null mutants *de novo* confirms that the *PIFTC3*-containing 29-40k amplicon is not required for viability of the null mutants, consistent with the previously demonstrated ability to cure null mutants of *PIFTC3* episomes that had been introduced into the parasites before generating the null mutants (16). We conclude that overexpression of the *PIFTC3* gene promotes growth of the glucose transporter-deficient parasites, thus facilitating detection of larger null mutant colonies but not being required to obtain such mutants when selections are performed under optimal conditions. How amplification of the *PIFTC3* gene and overexpression of the PIFTC3 protein achieve enhanced growth of the null mutants is not clear, especially since the established function of PIFTC3 is as a component of intraflagellar transport (IFT) particles involved in trafficking of flagellar proteins (26).

### GT3 is the principal hexose transporter expressed in infectious-stage amastigotes.

The existence of 3 distinct, albeit closely related, glucose transporters in L. mexicana raises the possibility that each transporter isoform assumes distinct biological functions, and this hypothesis has now been confirmed. Previous and current work establishes that GT2 is the principal hexose transporter for insect-stage promastigotes (Fig. 5C to F) and that functional expression of this permease is greatly reduced in amastigotes (Fig. 5G). GT1 is a flagellar hexose transporter (27, 28) that appears to play a role in monitoring or sensing the glucose level available to promastigotes, but expression of GT1 is also strongly attenuated in amastigotes (21). In contrast, the mutant expressing only GT3 is able to survive inside murine macrophages (Fig. 4A) and to induce lesions in BALB/c mice (Fig. 4C) as well as wild-type amastigotes. In addition, *GT3[+]* mutants transport glucose as well as wild-type axenic amastigotes (Fig. 5G), and the GT3-2A or GT3-GFP fusion proteins can be readily detected in intracellular amastigotes. These observations support the notion that GT3 is the distinct hexose transporter for amastigotes and that its unique transport activity in this infectious life cycle stage allows parasites to import sufficient glucose into the cytosol to thrive inside host macrophages.

One notable result of the current studies is that GT3 is largely restricted to the ER in intracellular amastigotes by the C-terminal ER retention motif. It is possible that a small but undetectable proportion of GT3 does traffic to the cell surface, and this limited amount of surface permease may be sufficient to promote viability of amastigotes inside macrophages. Indeed, one possible explanation for the targeted trafficking of GT3 to the ER is that this compartment represents a storage reservoir for this permease, which would be released to the plasma membrane only in limited quantities. This explanation would be consistent with the model of McConville and colleagues (5) that amastigotes operate a stringent metabolic network optimized for the nutrient-restricted environment of the macrophage phagolysosome and therefore maintain a reduced uptake capacity for hexoses in this life cycle stage (29).

However, it is possible that the role of GT3 in intracellular amastigotes is to transport glucose across the ER membrane into the cytosol. Since glucose is thought to be in relatively low supply in the parasitophorous vacuole (8, 9), utilizing an alternative source of carbohydrates in the ER might be advantageous to amastigotes. How could the ER function as an alternative source of glucose? In both *Leishmania* (30) and higher eukaryotes, quality control of glycoprotein biosynthesis in the ER employs a deglucosylation/reglucosylation cycle in which glucose is removed from the core N-linked glycans of newly synthesized proteins and this sugar is selectively added back to proteins that have not yet folded properly, employing UDP-glucose as the activated precursor. This quality control cycle generates free glucose in the lumen of the ER, and it may be advantageous for glucose-limited intracellular amastigotes to salvage this glucose from that organelle rather than exporting the sugar as a waste product. ER-resident GT3 would allow this sugar to be translocated to the cytosol where it could be employed either as metabolic fuel or as a biosynthetic precursor. Furthermore, transport of glucose across the ER membrane of mammalian cells has been documented using glucose nanosensors (31), providing a well-established precedent that glucose transporters can function in the ER of eukaryotes.

The regulated trafficking of GT3 from the ER to the cell surface in promastigotes as cell density increases is also unanticipated and of interest. Neither glucose limitation nor glucose supplementation altered localization to either the ER or cell surface, nor did studies with conditioned medium identify depletion or accumulation of other factors that control trafficking of LmxGT3. Hence, relocalization of GT3 as cell density increases may be irreversible once induced. Other biologically important responses to cell density have been observed in related trypanosomatids, most notably the phenomenon of social motility that has been characterized in Trypanosoma brucei procyclic parasites (32) and the induction of tsetse fly infectious stumpy-form parasites at high parasite density within the mammalian bloodstream (33).

Overall, the studies reported here underscore the dominant role of GT3 in the infectious amastigote stage of the life cycle and in parasite virulence *in vivo* and reveal an unanticipated complexity for the expression and localization of this transporter throughout the parasite life cycle.

## MATERIALS AND METHODS

### Cell lines and cultures.

Promastigotes of the L. mexicana wild type (strain MNYC/BZ/62/M379) or various glucose transporter null mutants derived from this wild-type strain were grown at 26°C on RPMI 1640 medium supplemented with 10% heat-inactivated fetal bovine serum, 0.1 mM xanthine, and 5 µg/ml hemin (10). Axenic amastigotes were grown in RPMI 1640-based medium containing 25 mM 2-(*N*-morpholino)ethanesulfonic acid buffer plus 20% heat-inactivated fetal bovine serum, 20 mM glucose, 100 µM adenosine, 10 µM folic acid, and 5 µg/ml hemin, adjusted to pH 5.6 as described previously (34) and maintained at 32°C. Growth curves were performed and quantified on a hemacytometer (10). Bone marrow-derived macrophages from BALB/c mice were isolated, cultured, and infected as reported (10).

### Generation of null mutants by homologous gene replacement.

Null mutants expressing only a single glucose transporter gene, *GT1[+]*, *GT2[+]*, and *GT3[+]*, were prepared by deleting the other two glucose transporter genes as indicated below, and the Δ*gt1-3[A-]* null mutant was prepared by deleting all three glucose transporter genes as indicated below. Various glucose transporter null mutants were constructed using pX63HYG (35) or pX63PAC (36) expression vectors in which the hygromycin phosphotransferase (HYG) or puromycin acetyltransferase (PAC) markers were bounded by 0.5- to 1.8-kb 5′ and 3′ flanking regions of each GT. These flanking regions used to target each gene replacement were as follows: Δ*gt1-3*, upstream targeting sequence (US), 1.45 kb extending 36 bp into the *GT1* ORF; downstream targeting sequence (DS), 1.8 kb beginning 10 bp after the stop codon of *GT3*; *GT1[+]*, US, 1.85 kb initiating within the 3′ untranslated region (UTR) of *GT1* and extending 140 bp into the *GT2* ORF; DS, 1.8 kb beginning 10 bp after the stop codon of *GT3*; *GT3[+]*, US, 1.45 kb extending 36 bp into the *GT1* ORF; DS, 0.5 kb starting 200 bp after the stop codon of *GT2*.

Wild-type L. mexicana promastigotes were first transfected (37) with purified targeting construct from the pX63HYG vector and plated on semisolid agar plates containing 80 µg/ml hygromycin. The correct integrations in the heterozygous knockouts were verified by Southern blots probed with the *GT2* ORF, and then one such clone was subjected to a second round of transfection using the excised targeting construct from the pX63PAC vector and plated onto semisolid medium containing 50 µg/ml hygromycin and 50 µg/ml puromycin. Plates were incubated at 26°C for extended periods of time, ~3 weeks, and very small colonies were picked. Clones were expanded, and Southern blot analysis was employed to confirm that homologous recombination had correctly supplanted the wild-type GT alleles. The *GT2[+]* null mutant was made starting with the Δ*gt1* (16) null mutant by using two more drug resistance cassettes from the pX63PHLEO (36) and pX63BSD (38) vectors. These targeting vectors encompassed either the phleomycin or blasticidin S deaminase resistance genes bounded by the 5′ and 3′ flanking regions of the *GT3* ORF: US, 1.0 kb extending 950 bp into the ORF; DS, 1.8 kb beginning 10 bp after the stop codon. Colonies from two sequential rounds of transfection were picked and expanded, and Southern blot analysis using the *GT2* ORF as probe was employed to confirm the generation of *GT2[+]*. The Δ*gt1-3[A-]* null mutant, lacking the linear 29-40k amplicon, was complemented with the *GT2* ORF subcloned into the pX63PHLEO expression vector to generate the “add back” line. The Δ*gt1-3[A-]* null mutant was also supplemented with the *PIFTC3* ORF on two different episomes, the pXNG4 (39) and pX63NEO plasmid expression vectors, or by integrating this ORF into the rRNA locus using the pIR1SAT vector (40).

### Nucleic acid purification, blotting, and hybridization.

Genomic DNA was isolated from parasite lines, digested with restriction enzymes, blotted onto nitrocellulose, and hybridized to radiolabeled probes as described previously (11).

### Glucose uptake assays.

Uptake of glucose was monitored in promastigotes or axenic amastigotes as described previously (41), except that 100 µM or 500 µM d-[^14^C]glucose (Moravek Biochemicals, Inc.; d-[U-^14^C]glucose; specific activity, 248 mCi/mmol) was employed, as indicated in each figure. Measurements of glucose uptake at different pH values (Fig. 7) were performed as reported previously (42), except that 100 µM d-[^3^H]glucose (Moravek Biochemicals, Inc.; d-[2-^3^H(N)]glucose; specific activity, 21.2 Ci/mmol) was employed as the substrate. Protein determination for normalization was performed using the DC protein assay kit (Bio-Rad).

### qRT-PCR.

Purification of mRNA and quantitative real-time PCR (qRT-PCR) were performed as previously described (43), using the StepOne Plus real-time PCR system (Applied Biosystems). Specific primers used for the *PIFTC3* gene were PIFTC3-RTF1 (GCCGATCAACCATACGTACCT) and PIFTC3-RTR1 (CCGCGTAAGGCACATTCTT); for the *GT4* gene, primers were D2F-3 (CGGCAACCAGGTGGGCTACTCC) and D2R-4 (CTTGCAGTAGTCGAGGCGC). Primers for amplification of 18S rRNA used as internal controls were Lmj18S-RTF (ATCAAACTGTGCCGATTACGTCCC) and Lmj18S-RTR (CGCCTGTCCGATCACCTGTATTGC).

### Generation of tagged GT2 and GT3 fusion proteins.

For integration into the endogenous gene loci, the *GT2* and *GT3* ORFs were cloned using a forward primer containing an SfiIA site (underlined), 5′ GAGGCCACCTAGGCCATGAGCGACAAGTTGGAGGCGAAC 3′, and reverse primers with an SfiIB site, GT2-SfiB-R (5′ GAGGCCACGCAGGCCCTCAGCCCTGTTGCCGCTGAGCGA 3′) and GT3-SfiB-R (5′ GAGGCCACGCAGGCCCATTTCTTTCTTCCCGACG 3′) as previously described (28). The *GT2* and *GT3* 3′ untranslated regions (UTRs) immediately after the stop codon were cloned using forward and reverse primers containing SfiIC and SfiID sites, respectively (underlined): GT2, 5′ GAGGCCTCTGTGGCCGCAGAATTAGGAAGACGCTGCAC 3′ and 5′ GAGGCCTGACTGGCCCGAGGCACGTCACAATGAGACCAG 3′; GT3, 5′ GAGGCCTCTGTGGCCGTAATATTCCACGATTATACGCCGCTC 3′ and 5′ GAGGCCTGACTGGCCGGAAGTGGTCCTGCAAAACACG 3′. These four DNA fragments were used to generate the GT2::TaV2A::LUC::BSD::GT2[3′-UTR] and GT3::TaV2A::LUC::BSD::GT3[3′-UTR] fusions as previously described (28). For expression from an episomal vector, *GT2*::*TaV2A*::*LUC* and *GT3*::*TaV2A*::*LUC* fusion genes were PCR amplified from the constructs above and cloned into the pX63NEO-R1 vector previously described (44). All constructs were confirmed by sequencing at the OHSU Sequencing Core Facility. The linearized construct was transfected into wild-type L. mexicana promastigotes, and transgenic parasites containing the integrated transgene replacing one GT allele were selected on agar containing 10 µg/ml of blasticidin. The GT2::TaV2A::Luc or GT3::TaV2A::Luc fusion proteins were detected with anti-TaV2A antiserum and anti-Luc as described below.

Constructs encoding GT fusion proteins with GFP, influenza virus hemagglutinin epitope (HA_3_), or Tv2A-luciferase appended to the C terminus of the transporter were constructed in the pXG-′GFP+ or pX63NEO expression vectors, respectively, as described previously (21).

### Immunoblot assays.

Promastigotes and amastigotes (~2 × 10^6^) were lysed in lithium dodecyl sulfate sample buffer, incubated at 65° for 15 min, separated on a 4 to 12% NuPAGE Bis-Tris minigel (Life Technologies, Carlsbad, CA), and transferred onto a nitrocellulose membrane by electroblotting. Membranes were blocked with phosphate-buffered saline (PBS) containing 0.1% Tween 20 and 5% milk for 1 h, incubated with rabbit anti-2A (1:3,333 dilution; Millipore, Burlington, MA) and mouse anti-*Renilla* luciferase (1:1,000; Millipore) antibodies in blocking solution for 1 h, washed 5 times for 10 min with PBS containing 0.1% Tween 20, and then incubated with anti-mouse or anti-rabbit horseradish phosphatase-conjugated secondary antibody (Thermo Scientific Pierce, Pittsburgh, PA) at a 1:20,000 dilution in PBS, 0.1% Tween 20, 5% milk followed by washing with PBS plus 0.1% Tween 20 (5 times for 10 min). SuperSignal West Pico chemiluminescent substrate (Thermo Fisher) was used for detection, and an ImageQuant LAS 400 (GE Healthcare) scanner was employed to acquire luminescent images. Adobe Photoshop and Illustrator CS6 (Adobe Corporation, San Jose, CA, USA) were used to create image compositions.

### Immunofluorescence microscopy.

Promastigotes and axenic amastigotes were collected, washed once with PBS, and allowed to settle onto poly-l-lysine-treated coverslips for 20 min. Cells (including THP-1-derived macrophages and intracellular amastigotes) were fixed with methanol at −20°C for 10 min. Fixed cells were washed 3 times for 5 min each with PBS and then blocked with 5% normal goat serum for 20 min. Coverslips were incubated with primary antibodies for 1 h at room temperature in PBS followed by 5 PBS washes of 10 min each. Subsequently, coverslips were incubated with secondary antibodies for 1 h. After secondary antibody incubation, coverslips were washed as described above. Coverslips were mounted onto microscope slides using 4′,6-diamidino-2-phenylindole (DAPI) Gold Prolong reagent (Molecular Probes, Eugene, OR). Antibodies, dilutions, and sources are as follows: rabbit 2A (TaV2A), 1:200 (Millipore, Burlington, MA); mouse α-tubulin, 1:2,000 (Sigma-Aldrich, St. Louis, MO, USA); chicken GFP, 1:500 (Aves Labs, Tigard, OR); rabbit BiP, 1:500 (gift from J. D. Bangs, University at Buffalo School of Medicine and Biomedical Sciences, Buffalo, NY, USA); anti-rabbit Alexa 488, 1:1,000; anti-mouse Alexa 594, 1:1,000; and anti-rabbit Alexa 594, 1:1,000 (Molecular Probes); anti-chicken Alexa 488, 1:500, and anti-mouse Cy-5, 1:500 (Jackson ImmunoResearch, West Grove, PA); antihemagglutinin (HA) 16B12 monoclonal antibody, 1:1,000 (BioLegend, San Diego, CA).

Images were captured on a DeltaVision Image Restoration System (Applied Precision, Issaquah, WA) consisting of a Nikon Eclipse TE2000 microscope base and a mercury light source. Cells were imaged through a 60× 1.40-numerical-aperture (NA) Nikon objective using SoftWoRx acquisition software version 5.0.0-R6 (Applied Precision, Issaquah, WA). Images were deconvolved in SoftWoRx and then analyzed and processed using ImageJ (NIH, Bethesda, MD). Figures were constructed using Adobe Photoshop and Illustrator CS6 (Adobe Corporation, San Jose, CA, USA). Figures represent *z* axis projections of 2 to 3 image planes where the depth of each plane is 0.5 µm.

### Macrophage and mouse infections.

Murine primary bone marrow macrophages or differentiated THP-1 macrophages were infected with stationary-phase promastigotes as described previously (21). For quantification, intracellular parasites were counted by microscopic enumeration for at least 100 macrophages. For mouse infections, female BALB/c mice, about 6 weeks of age, were injected in one hind footpad with 5 × 10^6^ stationary-phase parasites of each line suspended in 20 µl sterile phosphate-buffered saline (PBS). Before injection, parasites of each line had been passaged previously through a mouse and harvested from incipient lesions, 4 weeks postinfection, by injecting the lesion with ~40 µl of sterile RPMI medium, followed by withdrawal of the injected medium. This lesion extract was subsequently cultured in RPMI 1640 medium for ~2 weeks, followed by storage as a frozen stabilate in liquid nitrogen. Parasites were thawed and cultured briefly to stationary phase immediately prior to experimental mouse infections. For each parasite line, 5 mice were infected, and lesion size was monitored weekly by measuring the width, top to bottom, of the infected footpad with calipers. The footpad was measured prior to injection with parasites, and that dimension was subtracted from each subsequent measurement to calculate change in footpad thickness. In addition, the width of the contralateral hind footpad was also determined over the time course of the experiment. Animal experiments were approved by the Institutional Animal Care and Use Committee of the Oregon Health & Science University.
